# Components of the Endogenous Cannabinoid System as Potential Biomarkers for Interstitial Cystitis/Bladder Pain Syndrome

**DOI:** 10.3390/diagnostics12010019

**Published:** 2021-12-23

**Authors:** Saki Sultana, Geraint Berger, Christian Lehmann

**Affiliations:** 1 Department of Pharmacology, Dalhousie University, Halifax, NS B3H 4R2, Canada; gr869131@dal.ca (G.B.); chlehmann@dal.ca (C.L.); 2 Department of Anesthesia, Pain Management and Perioperative Medicine, Dalhousie University, Halifax, NS B3H 4R2, Canada

**Keywords:** endocannabinoid system, interstitial cystitis, bladder pain syndrome, biomarker, cannabinoid receptor, endocannabinoid ligand, endocannabinoid enzyme

## Abstract

Interstitial cystitis/bladder pain syndrome (IC/BPS) is a chronic condition causing bladder pressure and pain. The condition is of unknown etiology and is often accompanied by other symptoms, including chronic pelvic pain, increased urinary urgency, and frequency. There is no definitive diagnosis for IC/BPS, and treatment options are currently limited to physical therapy and medications to help alleviate symptoms. The endogenous cannabinoid system (ECS) is an important regulator of numerous physiological systems, including the urinary system. Modulations of the ECS have been shown to be beneficial for IC/BPS-associated pain and inflammation in rodents. As an attempt to identify potential biomarkers for IC/BPS, we reviewed experimental studies where the components of the ECS have been quantified in experimental models of IC/BPS. Further investigations using well-defined animal models and patients’ data are required to obtain stronger evidence regarding the potential for ECS components to be definitive biomarkers for IC/BPS.

## 1. Introduction

Interstitial cystitis/bladder pain syndrome (IC/BPS) is a chronic condition causing pelvic pain, pressure, or discomfort that is perceived to be related to the urinary bladder, often accompanied by various other urinary symptoms, such as increased urinary urgency and frequency [1,2]. These symptoms present in the absence of infection or other identifiable causes, reflecting the idiopathic nature of the disorder [3,4]. Symptoms often overlap with other genitourinary disorders, including chronic urethral syndrome, overactive bladder, vulvodynia, and endometriosis, leading to complications in the evaluation and subsequent treatment [5,6]. IC can be ulcerative or non-ulcerative although the distinct pathophysiology and clinical presentations of these two sub-types are not properly defined yet [7,8]. IC predominantly affects females, with an estimated 5:1 female to male ratio [9], with approximately 2.7–6.5% of American women experiencing symptoms consistent with an IC diagnosis [10]. However, only 9.7% report receiving a diagnosis with IC [11]. The diagnosis of IC can be challenging to attain, with patients often presenting with a wide variety of symptoms, physical examination findings, and clinical test responses [12,13,14]. National Institute of Diabetes and Digestive and Kidney Diseases (NIDDK) has set specific guidelines for the diagnosis of IC/BPS; however, proper diagnosis of the condition essentially depends on the exclusion of other disorders and is subjected to the physician’s judgment in many cases [15,16]. The variability of symptoms and complexity of diagnosis often leads to misdiagnosis, underdiagnosis, and/or delayed diagnosis of IC/BPS, which may lead to delayed or inappropriate treatment regimens. The diagnosis of similar diseases with overlapping symptoms has previously been resolved through the use of biomarkers [17]. This review aims to assess the prospect of the components of the endogenous cannabinoid system (ECS) as diagnostic biomarkers for IC/BPS.

### 1.1. Pathophysiology of IC/BPS

The pathogenesis and etiology of IC/BPS are multifactorial [18,19]. Bladder epithelial dysfunction, neurogenic inflammation, mast cell activation, and autoimmunity are associated with the pathogenesis of IC/BPS [20]. Disruption of the bladder urothelial permeability (also known as leaky urothelium) is the most common feature in IC/BPS, but the mechanism of urothelial disruption is still unclear [18,21]. Urothelial disruption in inflammation and irritation can be caused by any bacterial or fungal infection, trauma/stress, foreign bodies, or chemicals [7,20]. The disrupted urothelial barrier facilitates the migration of urinary solutes from urine into the bladder wall and triggers the symptoms associated with IC/BPS. Leakage of urinary potassium into the suburothelium results in depolarization of nerves and muscles and consequently triggers tissue injury [19,22].

Activation and infiltration of mast cells in the urothelium is another common pathological feature in IC/BPS. Infiltration of eosinophils and T lymphocytes is observed as well, suggesting the possible immune dysregulation associated with IC/BPS. Activated mast cells secrete cytokines and several proinflammatory and nociceptive mediators, including substance P and nerve growth factor (NGF), which are associated with the proliferation of nerve fibers [16,23].

Abnormal expression of cell adhesion proteins (uroplakins, chondroitin sulfate), tight junction protein zonula occludens-1 (ZO-1), E-cadherin, and bacterial defense molecule (GP51) in the bladder tissues of patients with IC/BPS strongly suggest abnormal differentiation in the bladder with IC/BPS [18,23]. Aberrant differentiation in the bladder urothelium observed in IC/BPS results in the altered synthesis of several proteoglycans [24,25]. Increased apoptosis and elevated E-cadherin expression in the bladder of IC/BPS patients is indicative of chronic inflammation associated with IC/BPS [25,26]. Upregulation of inflammatory mediators, especially TNF-α and p38 mitogen-activated protein kinase, are also linked to the increased apoptosis in the IC/BPS [7,26].

The bladder epithelial cells are acutely declined in patients with IC/BPS. This decrease in cellular proliferation may be caused by an antiproliferative factor (APF) produced by the bladder epithelial cells [27]. APF causes reversible inhibition of heparin-binding epidermal growth factor-like (HB-EGF-like) growth factor production and normal epithelial cell proliferation [26,27].

### 1.2. Biomarkers for IC/BPS

The lack of consensus on the pathophysiology of IC/BPS and the presence of overlapping syndrome among IC/BPS and other urinary disorders emphasize the urgency of the identification of a biomarker for proper diagnosis, prognosis, and treatment of this painful bladder condition. Investigation of potential biomarkers for IC/BPS has mostly been done focusing on the urothelial differentiation proteins, proteoglycan proteins, urinary nerve growth factors, cytokines, and chemokines [7]. Until now, a unique APF produced by the bladder epithelial cells and secreted in the urine of IC patients is the most promising urinary biomarker for IC/BPS [7,28,29,30,31]. APF is associated with the suppression of cell growth and differentiation and increases in transcellular permeability. In-vitro studies have demonstrated that cell proliferation and membrane integrity was restored upon removal of APF from the cell culture media [32]. APF also played a role in the therapeutic effect of hydrodistension in patients with IC/BPS [33].

Available evidence also suggests HB-EGF and EGF as potential markers for IC/BPS [7,33,34,35]. NGF also holds promise as a biomarker for IC/BPS as NGF is significantly upregulated in the bladder epithelial cells and urine of IC/BPS patients [7,36,37,38]. Among the cytokines and chemokines, urine IL-6, IL-8, and serum IL-6, IL-8, IL-1β, and TNF-α have shown promise [7]. However, a single cytokine in the urine or serum may not be a sole confirmation for the IC/BPS, as aberrant cytokine levels might be associated with other inflammatory conditions. A combination of several specific cytokines in the urine will be more appropriate for the confirmation of IC/BPS and differentiate it from other urinary conditions [7]. Determination of a genetic biomarker for the IC/BPS is in progress as well, which will shed light on the predisposing factors for IC/BPS [23,39,40].

### 1.3. The Endogenous Cannabinoid System

**Figure 1:**
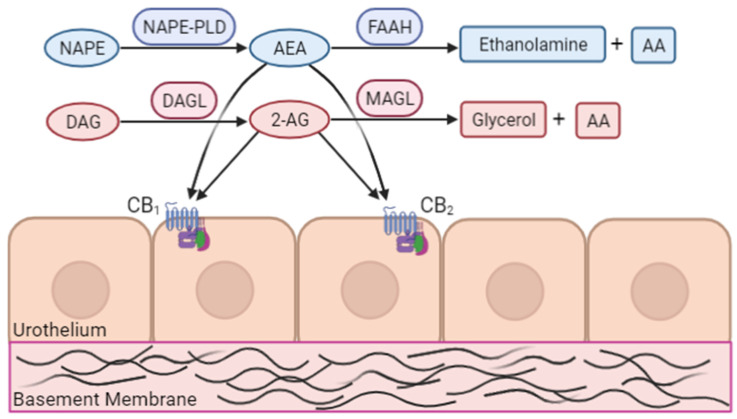
The endocannabinoid system (shown on urothelium) highlights key endocannabinoids, cannabinoid receptors, and synthesis/degradative enzymes. NAPE, N-acyl-phosphatidylethanolamine; NAPE-PLD, N-acyl-phosphatidylethanolamine-specific phospholipase D; 2-AG, 2-arachidonoylglycerol; AA, arachidonic acid; DAG, diacylglycerol; DAGL, diacyl-glycerol lipase; CB1, cannabinoid receptor 1; CB2, cannabinoid receptor 2; FAAH, fatty acid amide hydrolase; MAGL, monoacylglycerol lipase. (Image created with BioRender).

The endogenous cannabinoid system (ECS) is a biological lipid-based signalling system involved in a wide range of physiological functions, including but not limited to metabolism, neurotransmission, cognition, pain-sensation, and inflammation [41,42]. The discovery of endogenous bioactive lipids (endocannabinoids, ECs) that function through the same receptors that mediate the actions of tetrahydrocannabinol prompted the discovery of the ECS. Broadly, the ECS is comprised of three major components: the endocannabinoid signalling molecules/ligands, the enzymes involved in EC biosynthesis and degradation, and receptors upon which cannabinoid ligands function (Figure 1). Of the endocannabinoids, the most well studied are anandamide (AEA), and 2-arachidonylglycerol (2-AG), which are synthesized on-demand through enzymatic conversion of membrane lipids [43]. 2-AG is synthesized by the conversion of 1,2-diacylglycerol (DAG) to 2-AG by DAG-lipase [44] and AEA by conversion of N-arachidonylphosphatidylethanolamine (NAPE) by the enzyme phospholipase D [44]. Cannabinoid ligands function through membrane cannabinoid receptors 1 and 2 (CB1R and CB2R), which are G-protein coupled receptors expressed widely throughout the human body. The CB1R is predominantly expressed in the central nervous system, while the CB2R is localized to cells of the immune system and peripheral tissues [44,45]. CB1R accounts for the psychoactive property of cannabinoids, and CB2R is the key regulator of inflammation and immune regulation of cannabinoids. The ECS is a transiently active system, relying upon enzymatic degradation of ECs to terminate signalling [45].

### 1.4. The Endocannabinoid System and the Urinary System

In recent years, various studies have shown a strong link between the ECS and bladder physiology. The ECS can downregulate sensory bladder function during urine storage and micturition under normal physiological conditions [46,47]. Fatty acid amide hydrolase (FAAH), the EC-metabolic enzyme that degrades AEA, is expressed in the urothelium of the bladder in mice, rats, and humans [47,48]. CB1R and CB2R are present in the bladders of various species, including rodents [49,50], monkeys [51], and humans [52,53]. The expression of both receptors in the human detrusor and urothelium has been confirmed using real-time quantitative polymerase chain reaction (qPCR), immunohistochemistry, and western blot analysis [53]. Differential expression within the tissues of the bladder has been shown, with both receptors showing greater expression in the urothelium compared to the detrusor [52]. Urothelial cells express an abundance of CB2R [49,50,51]. Moreover, both receptors have been identified on afferent and cholinergic nerves within the bladder wall [54], where they co-localize with receptors involved in sensory and nociceptive signalling, including transient receptor potential vanilloid [55], transient receptor potential ankyrin 1 [56], and calcitonin gene-related protein [51].

Modulations of the ECS have been proven to be beneficial in urinary diseases, including lower urinary tract symptoms in painful bladder syndromes (PBS) and overactive bladder (OAB) [46,47,57]. Activation of the CB2R resulted in suppression of the sensitizing effects of the nerve growth factor on afferent nerve signaling and blocked increased peripheral mechanical sensitivity associated with inflammatory cystitis in mice [50,58]. Pharmacological inhibition of FAAH attenuated hyper-reflexia in inflamed rat bladders [59]. FAAH inhibition also improved bladder storage capacity, reduced frequency and residual volume, and increased threshold pressure in overactive bladders in rats [47]. The endocannabinoid ligand AEA inhibited nerve-mediated contractions in rodent bladders in a CB1R-dependent manner [47,60]. The effect of AEA on bladder contractions was lower in rats compared to mice, indicative of interspecies variation in AEA-dependent contractions in bladder tissue [47,60]. Systemic treatment of rats with cyclophosphamide-induced bladder inflammation with AEA or PEA attenuated viscero-visceral hyperreflexia [61]. AEA and PEA also prevented hyperalgesia associated with urinary bladder inflammation in rats [59,62]. Taken together, it is evident that the ECS has a strong regulatory effect on the urinary system.

We have reviewed the studies investigating the ECS in experimental animal models of IC/BPS and documented the changes in the levels of individual components of the ECS to determine whether any component of the ECS can serve as a potential biomarker to aid in the diagnosis of IC/BPS.

## 2. Search Strategy for the Review

PubMed, ScienceDirect, and Google Scholar databases were searched using the following keywords in various combinations: endocannabinoid system, endocannabinoids, interstitial cystitis, bladder pain syndrome, cannabinoid receptors, fatty acid amide hydrolase, monoacylglycerol lipase, anandamide, 2-arachidonoylglycerol, and biomarker. All studies up to 30 August 2021 were examined for the selection process. A manual search of references was performed on the bibliography of each selected article to further identify studies not captured by the above-mentioned keywords. Only articles published in English were considered.

## 3. Results

### 3.1. Cannabinoid Receptor Expression in IC/BPS

We identified seven studies where the expression of CB1R and/or CB2R was detected either at mRNA (RT-PCR/q-PCR) or protein level (western blot analysis or immunofluorescence staining) in experimental animal models of IC/BPS (Table 1). The CB1R expression (mRNA or protein) remained unchanged in all the relevant studies except the study by Pessina et al., where CB1R mRNA was upregulated in a cyclophosphamide (CYP)-induced cystitis model using female Wistar rats [63]. The upregulation of CB1R in this study could be model-dependent. On the other hand, the CB2R level was either upregulated or remained unchanged in experimental models of IC/BPS. Merriam et al. reported a significant increase of CB2R protein in the detrusor in acute cystitis models and a significant increase of CB2R protein both in the detrusor and mucosa in chronic cystitis in rats having two different backgrounds (Sprague–Dawley and Wistar) [49]. No change in the CB2R expression reported by some studies could be associated with the models used for those studies. As a good number of studies reported significant upregulation of CB2R in animal models of IC/BPS, CB2R appears to be promising as a biomarker for IC/BPS. This is a subject of further investigation whether there is any correlation between the upregulation of CB2R and disease severity in IC/BPS.

### 3.2. Endocannabinoid Ligands and Fatty Acid Ethanolamides in IC/BPS

We found five studies reporting the levels of endocannabinoid ligands and fatty acid ethanolamides in experimental models of interstitial cystitis (Table 2). Fatty acid ethanolamides are endocannabinoid-like compounds that interact with receptors outside of CB1R and CB2R [66]. Palmitoylethanolamide (PEA) was significantly increased in the bladder tissue of all the relevant studies except the study by Charrua et al., where PEA was downregulated in an LPS-induced cystitis model using Wistar rats [67]. The time-point for measuring the PEA content of the study by Charrua et al. was 24 h, which was different from the other two studies reporting higher levels of PEA in experimental IC/BPS. Merriam et al. reported the bladder PEA content after 48 h of instillation of acrolein [68], and Pessina et al., measured the PEA level after 3.5 h of intraperitoneal injection of CYP in rats [63]. Pessina et al., also reported the protective role of PEA in CYP-induced cystitis in rats, where intraperitoneal administration of ultra-micronized PEA attenuated pain behavior, voiding episodes, and bladder inflammation associated with CYP administration in female Wistar rats. This beneficial role of PEA in cystitis models indicates that elevation of PEA level is a defensive response of bladder to counteract local inflammation, but further investigation is required to confirm this [63].

Three studies reported significant increases in the AEA content, and two studies reported no change in the level of AEA in the bladder tissue of rats in experimental IC/BPS (Table 2). The experimental models of the studies in which the AEA level remained unchanged were different from the other studies reporting the level of AEA. Pessina et al. administered low-dose CYP (20 mg/kg) in rats [63], and Wang et al., used C57BL/6J mice for induction of cystitis [69], which could have possibly contributed to no detectable change in the bladder AEA content. Nonetheless, the underlying mechanisms for the diverging changes in PEA and AEA concentrations are unclear. The rates of synthesis and hydrolysis of the ethanolamides might be different in different models. Degradation of AEA and PEA by mechanisms other than hydrolysis may also play an important role in their balance.

2-AG and oleoylethanolamide (OEA) levels did not show any trend in the studies in which these two components were measured. One study reported a decrease in the level of 2-AG, whereas another study did not find any change in the bladder content of 2-AG. In the case of OEA, one study reported a significant increase in OEA, and another study showed no change in the level of OEA (Table 2).

As PEA and AEA were mostly upregulated in experimental models of IC/BPS, these two components can be considered as candidates for diagnostic biomarkers for IC/BPS.

**Table 2:** Endocannabinoid Ligands and Fatty Acid Ethanolamides in the Bladder Tissue of Experimental IC/BPS Models.

Animals	Condition	Method of Induction of IC	Endocannabinoid Ligands and Fatty Acid Amides	Reference
Female Wistar rats	CYP-induced cystitis	CYP (20 mg/kg), i.p. injection	Increased level of PEA; No change in AEA level; Significant decrease in 2-AG level	[63]
Female Wistar rats	Acrolein-induced bladder inflammation/hyperalgesia	Acrolein (1 mM, 400-μL total volume), intravesical instillation	Significant increase in PEA, AEA, and OEA	[68]
Female Wistar rats	CYP-induced cystitis	Two schedules of i.p. injections of CYP: single injection (200 mg/kg) or three injections (75 mg/kg) each on days 1, 4, and 7.	Significant increase in AEA levels in both cases at each time points	[70]
Male FAAH KO and WT mice having C57BL/6J background	CYP-induced cystitis	CYP (150 mg/kg), i.p. injection	No change in the bladder content of AEA and 2-AG in both WT and FAAH KO	[69]
Female Wistar rats	LPS-induced cystitis	LPS (5 mg/kg), intravesical instillation	Decreased level of PEA; Increased level of AEA; No change in the level of OEA	[67]

### 3.3. Endocannabinoid Enzymes in IC/BPS

The levels of endocannabinoid enzymes have rarely been measured in experimental models of IC/BPS. Only two studies have reported the levels of FAAH and NAAA in experimental cystitis models, and FAAH and NAAA remained unaltered in both cases. (Table 3).

## 4. Discussion

In this review, we have summarized information on the changes in the levels of different components of the ECS in experimental models of IC/BPS as an attempt of identifying potential biomarkers for IC/BPS. Based on currently available data from various experimental studies, we identified CB2R expression, and tissue levels of PEA and AEA look promising as potential biomarkers for IC/BPS as these components were upregulated in the bladder of most of the pre-clinical models of IC/BPS.

Although the ECS has been identified as a potential therapeutic target for the treatment of IC/BPS [50,57,63,64,65,71], there are not enough studies in which the contents of different components of the ECS have been measured in experimental models of IC/BPS. Moreover, there are discrepancies in the results of the studies reporting ECS components in animal models of IC/BPS. One of the reasons for the discrepancies could be the variations in the animal models being used. The endpoint of the studies and analytical parameters are also variable, which makes it challenging to compare the findings of different studies. In the case of CB receptors, most of the studies measured the contents of CB receptors at mRNA levels and on the whole bladder of the animals. It would be very useful if more information were available on the expression of CB receptors at the protein level in different layers of the bladders in animal models.

Interstitial cystitis is a chronic condition, but the majority of the available data on the contents of individual components of the ECS are from acute IC/BPS models. We came across only one study where a chronic acrolein-induced cystitis model was used [49]. Information on the changes in the levels of components of the ECS using chronic animal models for IC/BPS would be more reliable to derive a conclusion with respect to their values as potential biomarkers for IC/BPS. Moreover, available animal models for studying IC/BPS are chemically induced models, which do not accurately recapitulate the disease from which patients experience. Data obtained from real patients would provide stronger evidence on the changes in the contents of the ECS in the bladder affected by IC/BPS. The study by Mukherji et al., examined the bladder tissue specimens obtained from patients with painful bladder syndrome (PBS) and idiopathic detrusor overactivity (IDO) and showed a significant increase in CB1R-immunoreactive nerve fibers in the suburothelium of PBS and IDO bladders and the detrusor layer of IDO [72].

The studies that we have come across have not reported the content of the components of the ECS in the plasma or urine of the animal models of IC/BPS. When it comes to the diagnosis of a disease, blood or urine samples are easily accessible compared to obtaining tissue samples from an organ like the bladder. The endocannabinoids are synthesized in the body on-demand, and the half-life of the endocannabinoids are very short (minutes). The altered levels of endocannabinoids or the endogenous ligands in the bladder tissue may not be reflected in blood due to their short half-lives and presence of various metabolic enzymes in the blood. Urine can be analyzed to check whether the changes in the tissue levels of the endocannabinoids or PEA or AEA are reflected in the urine. In a study involving bladder cancer patients, urine samples were analyzed for the contents of ethanol amides. That study reported upregulation of AEA, PEA, N-stearoylethanolamide, and N-linolenoylethanolamide in the urine samples of patients with bladder cancer [73]. NAAA level was also reported to be upregulated in the urine of bladder cancer patients in that study [73]. Similar studies should be conducted involving IC/BPS patients to obtain information about the contents of endocannabinoids or ECS-metabolizing enzymes in the urine of IC/BPS patients, which would be valuable in identifying a biomarker for IC/BPS in a non-invasive manner.

## 5. Conclusions

As studies have shown strong links between the ECS and bladder function both in normal and pathological conditions, there is a strong possibility that components of the ECS, especially the endocannabinoid ligands, fatty acid amides, and CB2R, can serve as biomarkers for IC/BPS. More studies using carefully designed disease models and clearly defined analytical parameters are required to obtain more convincing data on the components of the ECS in IC/BPS. Additionally, promising findings from pre-clinical studies should be followed by well-structured clinical studies to determine whether any component of the ECS can work as a diagnostic biomarker for IC/BPS.

## Figures and Tables

**Table 1:** Endocannabinoid Receptor Expression in the Bladder Tissue of Experimental IC/BPS models.

Animals	Condition	Method of Induction of IC	CB1R	CB2R	Reference
Female Wistar rats	CYP-induced cystitis	CYP (20 mg/kg), intraperitoneal (i.p.) injection	Upregulation of CB1R mRNA expression	No change in CB2R mRNA expression	[63]
Female C57BL/6NH mice	Acrolein-induced cystitis	Acrolein (1 mM, 150-μL total volume), intravesical instillation	Not reported	No change in CB2R abundance in urothelium/suburothelium (western blot analysis)	[50]
Male CD1 mice	Lipopolysaccharide (LPS)-induced cystitis	LPS (Doses 20 mg/kg and 25 mg/kg), i.p. injection	No change in CB1R mRNA expression	Increase in CB2R mRNA expression	[64]
Female C57BL/6J mice	CYP-induced cystitis	CYP (150 mg/kg), i.p. injection	Not reported	Significant increase in CB2R mRNA expression	[65]
Female C57BL/6NH mice	Acrolein-induced cystitis	Acrolein (1 mM, 150-μL total volume), intravesical instillation	Not reported	CB2R-like immunoreactivity in urothelium remained unchanged	[58]
Female C57BL/6 mice	CYP-induced cystitis	CYP (300 mg/kg) i.p. injection	No change in CB1R expression pattern (immuno-fluorescence staining)	Not reported	[55]
Female Sprague–Dawley and Wistar rats	Acrolein-induced acute and chronic cystitis	**Acute cystitis:** One dose of acrolein **Chronic cystitis:** Three doses of acrolein at 72-h intervals. Acrolein dose: (400 μL, 1 mM)	**Acute cystitis:** No significant change in CB1R protein; No change in CB1R mRNA expression **Chronic cystitis:** No change in CB1R protein	**Acute cystitis:** Significant increase in CB2R protein in the detrusor but not in the mucosa; No change in CB2R mRNA expression **Chronic cystitis:** Significant increase in CB2R protein in detrusor and mucosa; CB2R mRNA increased	[49]

**Table 3:** Endocannabinoid Enzymes in the Bladder Tissue of Experimental IC/BPS Models.

Animals	Condition	Method of Induction of IC	Endocannabinoid Enzymes	Reference
Female Wistar rats	CYP-induced Cystitis	CYP (20 mg/kg), i.p. injection	No significant change in NAAA mRNA expression	[63]
Female Wistar rats	Acrolein-induced bladder inflammation/hyperalgesia	Acrolein (1 mM, 400-ul total volume), intravesical instillation	No change in FAAH protein expression	[68]

## Data Availability

Data sharing is not applicable to this article. No new data were created or analyzed in this study.

## Abbreviations

2-AG	2-Arachidonoylglycerol
AEA	Anandamide
APF	Antiproliferative factor
BPS	Bladder pain syndrome
CB1R	Cannabinoid receptor type 1
CB2R	Cannabinoid receptor type 2
CYP	Cyclophosphamide
EC	Endocannabinoid
ECS	Endogenous cannabinoid system
EGF	Epidermal growth factor
FAAH	Fatty acid amide hydrolase
HB-EGF	Heparin-binding epidermal growth factor
IC	Interstitial cystitis
IDO	Idiopathic detrusor overactivity
IL-6	Interleukin-6
IL-8	Interleukin-8
IL-1β	Interleukin-1β
TNF-α	Tumor necrosis factor- α
i.p.	Intraperitoneal
LPS	Lipopolysaccharide
MAGL	Monoacylglycerol lipase
mRNA	messenger ribonucleic acid
NAAA	N-acylethanolamine acid amide hydrolase
NGF	Nerve growth factor
OEA	Oleoylethanolamide
PEA	Palmitoylethanolamide
PBS	Painful bladder syndrome
qPCR	Quantitative polymerase chain reaction
